# Fatty Acid Conjugation Leads to Length-Dependent Antimicrobial Activity of a Synthetic Antibacterial Peptide (Pep19-4LF)

**DOI:** 10.3390/antibiotics9120844

**Published:** 2020-11-26

**Authors:** Philip Storck, Florian Umstätter, Sabrina Wohlfart, Cornelius Domhan, Christian Kleist, Julia Werner, Klaus Brandenburg, Stefan Zimmermann, Uwe Haberkorn, Walter Mier, Philipp Uhl

**Affiliations:** 1 Department of Nuclear Medicine, Heidelberg University Hospital, 69120 Heidelberg, Germany; philip.storck@med.uni-heidelberg.de (P.S.); florian.umstaetter@med.uni-heidelberg.de (F.U.); sabrina.wohlfart@med.uni-heidelberg.de (S.W.); christian.kleist@med.uni-heidelberg.de (C.K.); julia.werner@med.uni-heidelberg.de (J.W.); uwe.haberkorn@med.uni-heidelberg.de (U.H.); walter.mier@med.uni-heidelberg.de (W.M.); 2 Institute of Pharmacy and Molecular Biotechnology, Ruprecht-Karls-University, 69120 Heidelberg, Germany; Domhan@uni-heidelberg.de; 3 Brandenburg Antiinfektiva GmbH, Parkallee 10b, 23845 Borstel, Germany; kbrandenburg@fz-borstel.de; 4 Department of Infectious Diseases, Medical Microbiology and Hygiene, Heidelberg University Hospital, 69120 Heidelberg, Germany; stefan.zimmermann@med.uni-heidelberg.de

**Keywords:** synthetic antimicrobial peptides, fatty acid conjugation, antimicrobial activity, *Staphylococcus aureus*, *Enterococcus faecium*

## Abstract

The increasing number of infections caused by multidrug-resistant bacteria requires an intensified search for new antibiotics. Pep19-4LF is a synthetic antimicrobial peptide (GKKYRRFRWKFKGKLFLFG) that was previously designed with the main focus on high antimicrobial activity. The hydrophobic motif, LFLFG, was found to be essential for antimicrobial activity. However, this motif shows several limitations such as aggregation in biological media, low solubility, and small yields in peptide synthesis. In order to obtain more appropriate peptide characteristics, the hydrophobic motif was replaced with fatty acids. For this purpose, a shortened variant of Pep19-4LF (Pep19-short; GKKYRRFRWKFKGK) was synthesized and covalently linked to saturated fatty acids of different chain lengths. The peptide conjugates were tested with respect to their antibacterial activity by microdilution experiments on different bacterial strains. The length of the fatty acid was found to be directly correlated to the antimicrobial activity up to an ideal chain length (undecanoic acid, C11:0). This conjugate showed high antimicrobial activity in absence of toxicity. Time–kill studies revealed a fast and bactericidal mode of action. Furthermore, the first in vivo experiments of the conjugate in rodents demonstrated pharmacokinetics appropriate for application as a drug. These results clearly indicate that the hydrophobic motif of the peptide can be replaced by a single fatty acid of medium length, simplifying the design of this antimicrobial peptide while retaining high antimicrobial activity in the absence of toxicity.

## 1. Introduction

The synthetic anti-lipopolysaccharide peptide Pep19-2.5 (GCKKYRRFRWKFKGKFWFWG) was designed to bind and neutralize pathogen-associated molecular patterns (PAMPs) [1]. Sepsis is induced by PAMPs, including lipopolysaccharides (LPS) from Gram-negative bacteria and lipoproteins (LP) derived from Gram-positive bacteria [2]. These virulence factors represent the main components of bacterial cell membranes. They are released after cell death and lysis. Cell signaling pathways (e.g., NF-κB pathway) are induced upon mammalian receptor binding (CD14, Toll-like receptors TLR2 and TLR4) of these factors. This results in a strong cytokine secretion, damaging tissue and organs [3]. In this context Pep19-2.5 has been proven to bind and neutralize free LPS and LP in the bloodstream [1]; antiseptic/anti-inflammatory effects could also be demonstrated in vivo in mouse models [4]. Furthermore, Pep19-2.5, inhibits the effects of cytoplasmic LPS in human myeloid cells and keratinocytes [5].

Pep19-2.5 contains several positively-charged (basic) amino acids (arginine, lysine) that are combined with hydrophobic amino acids, mainly located at the C-terminus (tryptophan, phenylalanine). Hence, the positively-charged part of the peptide is known to interact with the carboxylate groups of the oligosaccharide backbone and the phosphate groups of LPS, whereas the hydrophobic part interacts with the hydrocarbon chains of LPS [3,6]. Despite the high neutralization of free PAMP motifs in the bloodstream, Pep19-2.5 showed only low antimicrobial activity [4]. Thus, Pep19-2.5 was further optimized to Pep19-4LF (GKKYRRFRWKFKGKLFLFG), resulting in a stronger antibacterial activity [7]. In one further study, a concentration-dependent antibacterial activity against biofilms on several clinically-relevant bacteria could be demonstrated for both peptides. This may allow for further applications such as implant functionalization, which could probably inhibit biofilm formation [8]. The potential of Pep19-2.5 and the structurally-related compound Pep19-4LF for their therapeutic application in bacterial skin infections was further investigated [9]. For this reason, this antimicrobial peptide was formulated in a cream for topical application [10]. It was furthermore reported that the optimization of the hydrophobic part of Pep19-2.5 led to a huge amount of different peptides with a high number of variable (hydrophobic) amino acid sequences [3]. However, slight changes in this hydrophobic motif also led to strong increase of MIC values. This implies that the design of an antibacterial peptide based on a specific sequence is a process difficult to manage [7]. The LFLFG motif of Pep19-4LF also showed some limitations with respect to the peptide synthesis including small peptide yields due to low solubility in aqueous media before peptide purification. Therefore, it was important to improve the peptide characteristics while remaining high antimicrobial activity.

**Figure 1:**
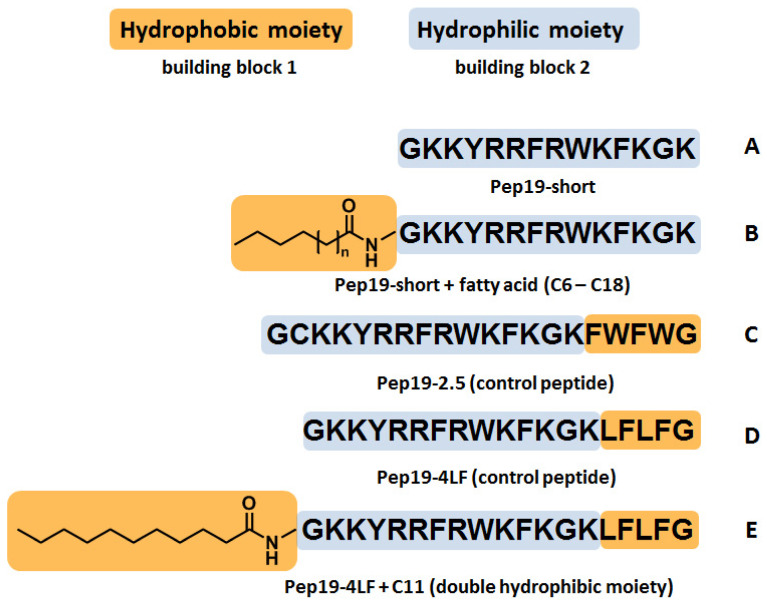
Schematic illustration of peptides synthesized and tested for antimicrobial activity by microdilution experiments: (**A**) Pep19-short, the peptide without a hydrophobic moiety, (**B**) Pep19-short conjugated to fatty acids including C6:0 (caproic acid), C8:0 (caprylic acid), C10:0 (capric acid), C11:0 (undecanoic acid), C12:0 (lauric acid), C14:0 (myristic acid), C16:0 (palmitic acid), and C18:0 (stearic acid), (**C**) Pep19-2.5 (original peptide), (**D**) Pep19-4LF (parent peptide), and (**E**) Pep19-4LF conjugated to undecanoic acid (C11:0) for an additional hydrophobic moiety.

To simplify the design and synthesis of the antimicrobial peptide, the hydrophobic motif (LFLFG) of Pep19-4LF was replaced by conjugation of different saturated fatty acids as a new lipophilic moiety (Figure 1). Fatty acids are (depending on the chain length) amphipathic molecules with a hydrophilic head group (carboxyl group) and a hydrophobic tail consisting of a variable number of alkyl groups. Previously, fatty acids were reported to show antimicrobial activity, e.g., by maintaining an acidic pH on the skin to impair the bacterial growth environment [11]. More importantly, they have been used to improve the characteristics of several antibiotics such as daptomycin [12] or dalbavancin [13]. The novel peptide conjugates synthesized in this project were characterized with respect to their antimicrobial activity against Gram-positive (*Staphylococcus aureus*; ATCC 25923 and *Enterococcus faecium*; UL602570*) and Gram-negative bacteria (*Acinetobacter bohemicus*; DSM 100419). Additionally, MBC values were determined for Gram-negative *Acinetobacter bohemicus* (DSM 100419) and Gram-positive *Rothia kristinae* (DSM 20032). A time–kill study was performed on *Rothia kristinae* for the most potent conjugate, C11-Pep19-short, in comparison with the parent peptide Pep19-4LF. Biodistribution studies were performed in Wistar rats for Pep19-4LF, Pep19-short, and C11-Pep19-short and revealed pharmacokinetics appropriate for application as a drug.

## 2. Results

### 2.1. Antimicrobial Activity

**Figure 2:**
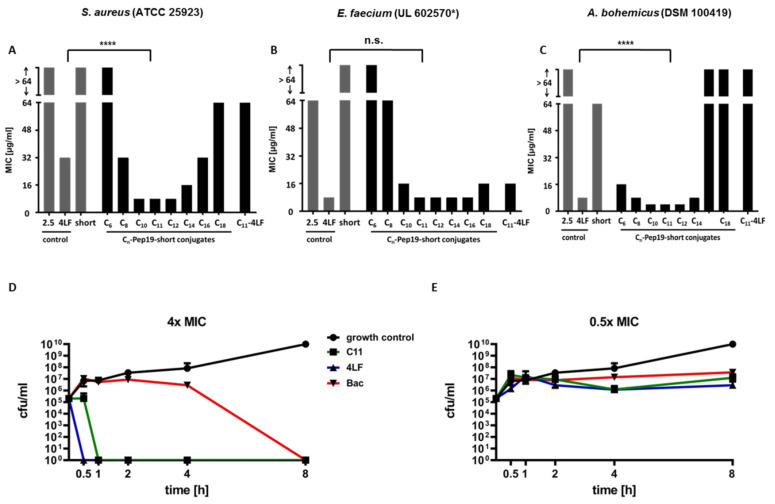
MIC values of Pep19-short conjugated to different fatty acids on (**A**) *S. aureus* (ATCC 25923), (**B**) *E. faecium* (UL602570*, clinical isolate, vanA-resistant), and (**C**) *A. bohemicus* (DSM 100419) and time–kill studies of the most potent fatty acid conjugate (C11-Pep19-short) on *Rothia kristinae* (DSM 20032) for (**D**) 4 × MIC and (**E**) 0.5 × MIC (n = 3). The length of the conjugated fatty acid is crucial for the antimicrobial activity of the peptide conjugates against all bacterial strains. Short fatty acids (C6:0, C8:0) did not show antimicrobial activity on *S. aureus* (**A**) or *E. faecium* (**B**) and only moderate antimicrobial activity on *A. bohemicus* (**C**). In contrast, fatty acids of medium length showed the highest antimicrobial activity with minimal inhibitory concentrations (MICs) at least comparable to or even lower than those of the control peptide, Pep19-4LF. Longer fatty acids showed lower antimicrobial activities resulting in higher MICs. Time–kill studies showed a bactericidal activity of C11-Pep19-short, Pep19-4LF and bacitracin at a concentration of 4 × MIC (**D**). As expected, a concentration of 0.5 × MIC was not high enough for an effective antimicrobial activity (**E**). For statistical analysis, a one-way ANOVA followed by a Turkey’s comparison test was applied. The significance is given by **** *p* < 0.0001.

MIC values were determined to investigate the antimicrobial potential of the conjugates on different bacterial strains. Figure 2 represents the Minimal Inhibitory Concentration (MIC) values of Pep19-short, Pep19-2.5, Pep19-4LF, Cn-Pep19-short (n = length of fatty acid), and C11-Pep19-4LF on Gram-positive (A) *Staphylococcus aureus* (ATCC 25923), (B) *Enterococcus faecium* (UL602570*, clinical isolate, vanA-resistant), and Gram-negative (C) *Acinetobacter bohemicus* (DSM 100419) as well as time–kill studies on Gram-positive *Rothia kristinae* (DSM 20032) for the most potent fatty acid conjugate (C11-Pep19-short) at a concentration of 4 × MIC (D) and 0.5 × MIC (E). For MIC values on Gram-positive *Rothia kristinae* used for the determination of MBC values as well as the time–kill studies, see Supplementary Materials. Time–kill studies were performed for Pep19-4LF, C11-Pep19-short, and bacitracin as control for a period of 0 to 8 h.

The MIC value of Pep19-4LF was determined at 32 μg/mL (*S. aureus*, Figure 2A), 8 μg/mL (*E. faecium*, Figure 2B), and 8 µg/mL (*A. bohemicus*, Figure 2C), respectively. Removal of the LFLFG motif caused a strong increase in the MIC values (>64 μg/mL) for *S. aureus*, *E. faecium* (Figure 2A,B), and *A. bohemicus* (64 µg/mL). Peptide conjugates with short fatty acids (C6:0, C8:0) also showed higher MIC values (*S. aureus*: >64 μg/mL (C6:0)/32 μg/mL (C8:0); Figure 2A and *E. faecium*: >64 μg/mL (C6:0)/64 μg/mL (C8:0); Figure 2B). For *A. bohemicus*, a stronger decrease of the MIC value was observed (16 µg/mL (C6:0)/8 μg/mL (C8:0); Figure 2C). Fatty acids of medium chain length provided a plateau of MIC values that were either equal or even lower when compared to the original peptide, Pep19-4LF (*S. aureus*: 8 μg/mL for C10:0, C11:0, C12:0; Figure 2A, *E. faecium*: 8 μg/mL for C11:0, C12:0, C14:0; Figure 2B, and *A. bohemicus* 4 µg/mL for C10:0, C11:0, C12:0, Figure 2C). Longer fatty acids (C16:0, C18:0) instead again provided higher MIC values, especially for *S. aureus* and *A. bohemicus* (Figure 2A,C). In the case of *E. faecium*, the shift to higher MICs for longer fatty acids was not as high as for *S. aureus* and *A. bohemicus*. With respect to these results, undecanoic acid (C11:0) was identified as the conjugated fatty acid providing the highest antimicrobial activity. Besides, the conjugation of undecanoic acid to the full-length peptide Pep19-4LF showed no further decrease of MIC.

Time–kill studies revealed a fast and bactericidal mode of action against *Rothia kristinae* for C11-Pep19-short. At a concentration of 4 × MIC all bacteria were killed within 30 min (Figure 2D). Pep19-4LF also showed a bactericidal mode of action for 4 × MIC (no detectable number of cfu after 30 min). As expected, a concentration of 0.5 × MIC showed only a small decrease in the number of cfu/mL. The control antibiotic bacitracin also showed a bactericidal activity at a concentration of 4 × MIC (no detectable cfu after 8 h) and only a small decrease in the number of cfu/mL for 0.5 × MIC. For further concentrations (1 × MIC and 2 × MIC), see Supplementary Materials.

### 2.2. Hemolysis Assay and Cytotoxicity Assay

**Figure 3:**
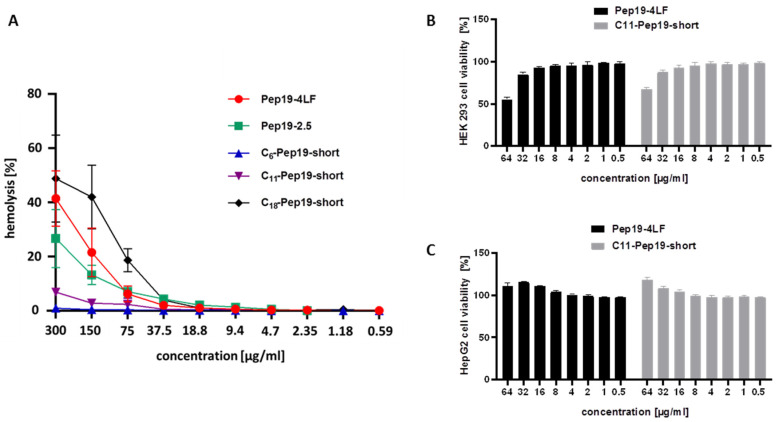
(**A**) Hemolysis study for Pep19-4LF, Pep19-2.5, C6-Pep19-short, C11-Pep19-short, and C18-Pep19-short as well as cytotoxicity assay for Pep19-4LF and C11-Pep19-short on HEK 293 cells (**B**) and HepG2 cells (**C**) (n = 3). C6-Pep19-short showed no hemolytic activity at all concentrations measured. C11-Pep19-short showed only low hemolytic activity at a concentration higher than 75 µg/mL. However, this was lower compared to Pep19-4LF, Pep19-2.5, and C18-Pep19-short. Pep19-4LF and C11-Pep19-short had no cytotoxic effects on both cell lines at MIC-relevant concentrations.

A hemolysis assay was performed to exclude toxicity of the peptide conjugates against red blood cells. (Figure 3A)

All peptides showed no hemolytic activities in MIC-relevant concentrations. For high concentrations of ≥150 µg/mL, the modified peptide C11-Pep19-short showed lower hemolytic activity compared to the parent peptides, Pep19-4LF and Pep19-2.5, indicating improved characteristics of C11-Pep19-short. Additionally, cytotoxicological examination of liver and kidney cells was performed at concentrations from 0.5 µg/mL to 64 µg/mL (Figure 3B,C). Cytotoxicity was only detected on kidney HEK 293 cells but at concentrations above the MIC values. In contrast, both peptides C11-Pep19-short and Pep19-4LF had no cytotoxic effects upon HepG2 liver cells. 

### 2.3. Biodistribution in Wistar Rats

**Figure 4:**
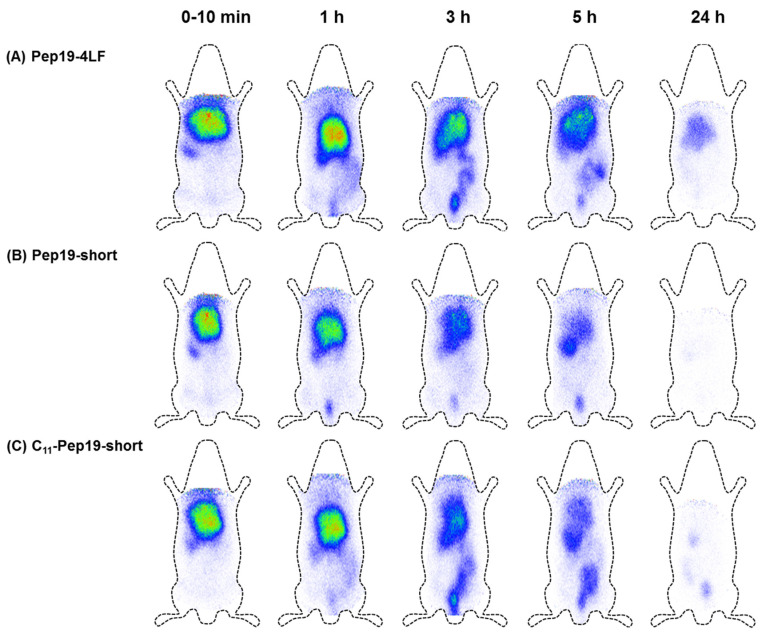
Scintigraphical images of (**A**) Pep19-4LF, (**B**) Pep19-short, and (**C**) C11-Pep19-short. The peptides were labeled with ^125^I and injected intravenously into the tail vein of female Wistar rats, the images were recorded 0–24 h post administration. All peptides showed comparable pharmakokinetics with fast liver enrichment and subsequent hepatobiliary excretion.

To investigate the biodistribution of Pep19-4LF, Pep19-short, and C11-Pep19-short, the peptides were labeled with the radioisotope ^125^I and injected in the tail vein of adult female Wistar rats. For the radio-HPLC diagrams of purified ^125^I-labeled Pep19-4LF and C11-Pep19-short, see Supplementary Materials. Figure 4 shows the scintigraphic images of Pep19-4LF (A), Pep19-short (B) and C11-Pep19-short (C) at determined time points. All peptides used for radiolabeling were synthesized with D-tyrosine instead of L-tyrosine to achieve higher stability of the introduced radioactive iodine.

The scintigraphic images indicated a high accumulation of all peptide-conjugates in the liver within the first 10 min. A small amount could also be detected in the spleen. The elimination occurred via the gastrointestinal tract within 24 h. The slowest elimination was observed for Pep19-4LF (A). C11-Pep19-short showed pharmacokinetic properties similar to Pep19-4LF. However, a faster excretion from the body was achieved which might decrease potential side effects. Despite the conjugated fatty acid in C11-Pep19-short, there was no difference in the elimination time when compared to Pep19-short without a fatty acid. In general, distribution and route of elimination were comparable between all peptide variants. Additionally, we investigated the liver metabolism of C11-Pep19-short. In this study, a half-life of about one hour was determined (for data, see Supplementary Materials).

## 3. Discussion

The high MIC value of Pep19-short indicates the importance of a hydrophobic moiety for this synthetic antimicrobial peptide. In this study we could show that the hydrophobic moiety can be replaced by a fatty acid. However, the length of the fatty acid was found to be crucial for the antimicrobial activity of the peptide conjugates [11]. If the length of the fatty acid is too short, the interaction between the peptide conjugate and components of the bacterial cell membrane might be unfavorable, thus leading to loss of efficiency. Fatty acids of medium chain length enable a stable plateau of low MIC values, indicating a more efficient interaction between the peptide conjugate and bacterial cells. Longer fatty acids also show a tendency for higher MIC values. A detailed interaction between Pep19 peptides and membrane-bound toxins was previously described by Correa et al. [7]. In this study, atomic force microscopy showed a change in bacterial morphology after treatment with Pep19-4LF, indicating a direct interaction between the peptide and bacteria. A hypothesized mechanism for this interaction represents the reorganization of the bacterial membrane leading in an inability to maintain the osmotic pressure. Here, the positively-charged amino acids of the peptide conjugate might interact with the negatively-charged compounds of the bacterial cell wall and the hydrophobic part interacts with hydrophobic lipids of the bacterial membrane. Several mechanisms for a membrane disruption by amphiphilic peptides were further described by Sato and Feix [14]. With respect to the parent peptide Pep19-4LF, the hydrophobic moiety was located at the C-terminus. As the synthesis leads to peptides that are linked to the resin via their C-terminus, the fatty acids were linked to the free N-terminus of the peptide. However, this change of the lipophilic part did not influence the antimicrobial activity. As comparison, coupling was also performed on the C-terminal side, leading to comparable MIC values on all tested strains. However, the synthesis was more complicated. Thus, N-terminal modification is preferred. Moreover, the conjugation of fatty acids to the N-terminal side also improved the peptide synthesis. All peptide conjugates showed a higher solubility in aqueous solution compared to Pep19-4LF. This was especially the case for the conjugation of shorter fatty acids (including undecanoic acid, C11:0). After peptide synthesis, Pep19-4LF showed a very low solubility in water and had to be filtrated before purification by preparative HPLC. As a consequence, a high loss of peptide was obtained, resulting in a total yield of 16.2%. In contrast, C11-Pep19-short showed high solubility in water and therefore, filtration before purification was not necessary. This led to a significantly-higher peptide yield of 32%. In general, the results demonstrate that modification of antibacterial peptides with fatty acids can be a tool for improving the characteristics of antimicrobial peptides [15,16]. According to Galbraith et al., saturated fatty acids with 10 or 12 carbon atoms in chain length in general show the highest antimicrobial efficacy [17]. These findings are in accordance with the results of this study and several studies published previously [18,19,20]. Longer fatty acids were found to form aggregates leading to a reduced antibacterial activity [16]. Time–kill studies revealed a bactericidal mode of action of Pep19-4LF and C11-Pep19-short. This was also confirmed by the determination of MBC values (see Supplementary Materials). Furthermore, all peptides showed no hemolytic activities and cytotoxic effects in MIC-relevant concentrations. For high concentrations (≥150 µg/mL), C11-Pep19-short showed lower hemolytic activity when compared to the parent peptide, Pep19-4LF. This shows an improvement of the modified peptide. In general, the hemolytic activity correlated with the length of the conjugated fatty acid [16]. According to Jannadi et al., Pep19-2.5 and Pep19-4LF were also nontoxic to monocytes, indicating a low risk of toxic events [21]. In line with these findings, in our study, C11-Pep19-short and Pep19-4LF also revealed no toxicity upon liver and kidney cells in MIC-relevant concentrations. This is important for potential efficacy studies in vivo, since rodent studies revealed high accumulation of the peptides in the liver.

Investigation of potential metabolism revealed that the in vivo half-life of C11-Pep19-short is in the range of about 1 h. Previous studies for the parent peptide Pep19-2.5 demonstrated a rapid loss of the free peptide [4]. This was also observed for C11-Pep19-short. An attachment to plasma components was assumed to be the reason for these findings. However, preclinical studies for Pep19-2.5 showed that the efficacy of the compound in vivo was not limited by this effect [4]. Previous in vitro studies performed by Correa et al. also showed a significant reduction of cytokines by Pep19-2.5 using a whole blood assay [7]. Despite the high plasma binding, these results demonstrated the effectiveness of the investigated peptides. 

The first in vivo studies by radiolabeling and subsequent molecular imaging demonstrated a high liver accumulation for both the modified and the unmodified peptide. The faster liver elimination of C11-Pep19-short compared to Pep19-4LF might decrease potential side effects. A high liver accumulation often goes along with a rapid clearance of the blood stream. Therefore, further optimization of the peptide modification could be performed by the introduction of PEG moieties to provide prolonged plasma half-life.

## 4. Materials and Methods 

### 4.1. Synthesis of Peptide Conjugates

Pep19-short (GKKYRRFRWKFKGK-NH_2_; MW = 1884.13 g/mol), Pep19-2.5 (GCKKYRRFRWKFKGKFWFWG-NH_2_; MW = 2710.46 g/mol), Pep19-4LF (GKKYRRFRWKFKGKLFLFG-NH_2_; MW = 2463.02 g/mol), Cn-Pep19-short (n = length of fatty acid), and C11-Pep19-4LF (MW = 2629.76 g/mol) were synthesized by solid-phase peptide synthesis (SPPS) applying the Fmoc/tBu strategy in an automated solid-phase peptide synthesizer (ABI 433A, Applied Biosystems, Thermo Fisher Scientific, Darmstadt, Germany) according to Schieck et al. [22]. All peptides were synthesized on a Tenta Gel R RAM resin (Rapp polymers, loading 0.2 mmol/g). Fmoc-L amino acids were purchased from Orpegen Peptide Chemicals GmbH, Heidelberg, Germany. After peptide synthesis, the fatty acids were coupled manually to the N-terminus of Pep19-short. For this purpose, 10 equivalents of the respective fatty acid (C6:0-C18:0), 9.5 equivalents of 2-(1*H*-benzotriazol-1-yl)-1,1,3,3-tetramethyluronium-hexafluorophosphat (HBTU) (Iris Biotech GmbH, Marktredwitz, Germany), and 20 equivalents of diisopropylethylamine (DIPEA) (Biosolve, Dieuze, France) were mixed with the resin in *N*-methyl-2-pyrrolidon (NMP) (Iris Biotech GmbH (Marktredwitz, Germany) and reacted for 3 h. As a next step, the peptide conjugate was cleaved from the resin with a mixture of 95% trifluoroacetic acid (TFA) (Biosolve, Dieuze, France), 2.5% triisopropylsilane (TIS) (Sigma-Aldrich, Steinheim, Germany), and 2.5% H_2_O.

Undecanoic acid (C11:0) was further coupled to the C-terminal side of Pep19-short. Therefore, an allyloxycarbonyl (alloc)-protected lysine (Iris Biotech GmbH, Marktredwitz, Germany) was introduced at the C-terminal side of the peptide. In the first step after peptide synthesis, the alloc protective group was cleaved for 20 min by using 4 mg Tetrakis(triphenylphosphine)palladium(0) (Sigma-Aldrich, Steinheim, Germany) and 40 mg borane–dimethylamine complex (Sigma-Aldrich, Steinheim, Germany), dissolved in 2 mL dichlormethane. Next, three washing steps in dichlormethane and methanol were performed. To remove all further impurities, the resin was washed with 2 mL 10:1 dichlormethane/methanol for 2 × 30 min and dried under vacuum. Finally, the fatty acid was coupled as described above. After the conjugation step, the peptide was cleaved from the resin with 95% TFA, 2.5% TIS, and 2.5% H_2_O for 2 h.

The successful coupling was verified by high-performance liquid chromatography (HPLC, Agilent 1100 Series, Chromolith^®^ Performance RP-18e, 100-3 mm column) and HPLC-MS (ESI-Orbitrap; Exactive, Thermo Fisher Scientific). Afterwards, the peptide conjugate was purified by preparative HPLC (Reprosil™ Pur 120 C18-AQ, 5 μm (250 × 25 mm) column) and the purity of the product was again analyzed by HPLC and HPLC-MS. Detailed MS-chromatograms of Pep19-short, C11-Pep19-short, Pep19-4LF, C11-Pep19-4LF, and Pep19-2.5 are implemented in the Supplementary Materials.

### 4.2. Antimicrobial Activity

#### 4.2.1. Minimal Inhibitory Concentration (MIC)

Minimal inhibitory concentrations (MICs) were determined against Gram-positive *Enterococcus faecium* vanA (UL 602570*, clinical isolate from Institute for Medical Microbiology and Hygiene, Heidelberg University Hospital, Heidelberg, Germany), *Staphylococcus aureus* (ATCC 25923), *Rothia kristinae* (DSM 20032), and Gram-negative *Acinetobacter bohemicus* (DSM 100419) (all obtained from the Department of Infectious Diseases, Medical Microbiology and Hygiene, Heidelberg University, Heidelberg, Germany) in a 96-well polypropylene plate (650261 Greiner Bio-One GmbH, Frickenhausen, Germany) according to European Committee on Antimicrobial Susceptibility Testing (EUCAST) and The Clinical & Laboratory Standards Institute (CLSI) guidelines [23,24]. Briefly, the stock solutions of the peptide conjugates in water were adjusted to a concentration of 1.28 mg/mL. Starting with the highest concentration of 64 μg/mL in 96-well polypropylene plates, dilution series in cation-adjusted Mueller–Hinton broth (MHB II; Sigma Aldrich, Steinheim, Germany) were performed, resulting in the lowest concentration of 0.125 μg/mL. A logarithmic growing bacterial culture was adjusted to a McFarland corresponding to 1 *×* 10^8^ cfu/mL using a McFarland counter (DensiCHEK^®^ plus, bioMerieux, Marcy-l’Étoile, France). Finally, a bacterial suspension of 1 × 10^6^ cfu/mL (further diluted in MHB II) was added to each peptide-conjugate concentration. Thus, the final bacterial concentration in each well was 5 × 10^5^ cfu/mL. The bacteria were incubated at 37 ± 1 °C (for *A. bohemicus*: 30 ± 1 °C) overnight and the bacterial growth was analyzed after 18 to 20 h. The MIC was defined as the lowest concentration of substance without any visible growth [23]. Vancomycin and colistin sulfate were purchased from Sigma-Aldrich Chemie GmbH, München, Germany and Carl Roth GmbH, Karlsruhe, Germany.

#### 4.2.2. Minimal Bactericidal Concentration (MBC)

Minimal bactericidal concentrations (MBCs) were determined by pipetting 3 µL of each broth dilution solution without visible growth onto a new agar plate. The agar plates were incubated at 37 ± 1 °C (for *A. bohemicus*: 30 ± 1 °C) for 24 h and were again analyzed with respect to bacterial growth. MBC is defined as the lowest concentration killing 99.9% of the inoculum [25]. 

#### 4.2.3. Time–Kill Studies

Time–kill studies were performed in accordance with CLSI guidelines [26]. Therefore, *Rothia kristinae* DSM 20032 was adjusted to a concentration of 1 x 10^8^ cfu/mL and further diluted to 1 × 10^6^ cfu/mL in cation-adjusted MHB II. According to the previous determined MIC values of Pep19-4LF, C11-Pep19-4LF, and bacitracin (positive control) (Sigma Aldrich Chemie GmbH, München, Germany), samples were taken for 4 × MIC, 2 × MIC, 1 × MIC, and 0.5 × MIC after 30 min, 1 h, 2 h, 4 h, and 8 h. These samples were diluted in sterile saline and 10 µL of each sample was loaded onto agar plates. A bacterial suspension of 1 × 10^6^ cfu/mL served as a growth control. Incubation occurred at 37 ± 1 °C and all counted colonies were recalculated to the colony number in the original inoculum. 

### 4.3. Hemolysis and Cytotoxicity Assay

A hemolysis assay was performed using heparinized blood for Pep19-4LF, Pep19-2.5, C6-Pep19-short, C11-Pep19-short, and C18-Pep19-short to test the toxicity against red blood cells and selectivity against bacterial cells. A 600 µM stock solution of each peptide was prepared in 0.9% NaCl. Heparinized blood was taken from three individual fasted, healthy volunteers. To purify the erythrocytes, the blood was centrifuged at 2500 rpm for 10 min and the supernatant was removed. The cell-pellet was resuspended in standard PBS. The centrifugation step was repeated for three times until the supernatant was visibly clear. A 96-well v-bottom plate (Greiner Bio-One International GmbH, Kremsmünster, Austria) was loaded as follows: row 1 (A1-H1) was preloaded with 100 µL of 600 µM peptide stock solution and row 12 (A12-H12) was preloaded with 50 µL 10% Triton X-100 (PerkinElmer, Boston, USA) and served as a positive control. All other wells were preloaded with 50 µL of PBS and dilution series of the peptide stock solutions (starting from column 1) were performed by transferring 50 µL peptide solution to the next well (until row 10). Row 11 (A11-H11) served as a negative control, containing only PBS. All substances were tested in duplicate in three individual assays starting with the highest concentration of 300 µM up to the lowest concentration of 0.59 µM. Finally, 50 µL purified erythrocytes were added to each well and the 96-well plate was incubated at 37 ± 1 ° C for 1 h. After incubation, 75 µL PBS were added to each well and the plate was centrifuged at 4000 rpm for 2 min. Fifty µL of the supernatant was then transferred to a new 96-well flat-bottom plate (Greiner Bio-One International GmbH, Kremsmünster, Austria) and the absorbance was measured at a wavelength of 554 nm, using an Infinite M200 PRO microplate reader (Tecan Trading, Maennedorf).

Hemolysis was calculated with the following equation:

$$Hemolysis\;\left[\%\right]=\;\frac{A_{conjugate}-\;A_{blank}}{A_{Triton}-\;A_{blank}}$$

To exclude potential toxicological effects of novel peptides, cytotoxicity studies of C11-Pep19-short and Pep19-4LF were performed using a colorimetric viability assay according to Umstätter et al. [27]. Therefore, human embryonic kidney cells (HEK 293) and human liver cancer cells (HepG2) were used. Briefly, cells were seeded on 96-well plates (Costar, Corning, Tewksbury, USA) at a concentration of 1.5 × 10^4^ cells/well and cultured overnight in a humidified atmosphere at 37 °C and 5% CO_2_. Subsequently, the peptides were added at concentrations ranging from 64 µg/mL to 0.5 µg/mL and incubated for 24 h. After an incubation time of 24 h, the XTT reagent (2,3-bis-(-2-methoxy-4-nitro-5- sulfophenyl)-2H-tetrazolium-5-carboxanilide salt) and the activation reagent phenazine (Applichem, Darmstadt, Germany) were added. Finally, the absorption was determined by measurements on an Infinite M200 PRO microplate reader at 470 nm and 670 nm (reference). Untreated cultured cells (=100% viability) were used as a control.

### 4.4. Digestion of C11-Pep19-Short with S9 Fraction from Human Liver

The digestion of C11-Pep19-short in S9 mix from human liver (Sigma Aldrich, Steinheim, Germany) was performed according to the manufacturer´s online protocol. In brief, a stock solution of the peptide conjugate (20 mg/mL) was prepared in 0.9% NaCl. Next, 183 µL of 0.9% NaCl and 2 µL of the peptide stock solution were pre-incubated for 5 min at 37 °C. Then, 5 µL of the 20 mg/mL S9 mix was added to the peptide solution. Next, 10 µL of 20 mM NADPH-solution (Sigma-Aldrich, Steinheim, Germany) was added and the mixture was shaken for 1 h at 37 °C. After the incubation time the reaction was stopped by the addition of 200 µL acetonitrile. The sample was centrifuged for 5 min at 10,000 rpm and the supernatant was analyzed by LC–MS.

### 4.5. In Vivo Experiments in Female Wistar Rats 

The animal experiments were approved by the Animal Care and Use Committee at Regierungspräsidium Karlsruhe (Karlsruhe, Germany; reference: G-111/16). Scintigraphic images were recorded for Pep19-4LF, Pep19-short, and C11-Pep19-short in female Wistar rats (200–250 g), purchased from Janvier Labs (Le Genest-Saint-Isle, France). For this purpose, the tyrosine residues of the peptides were radiolabeled with ^125^I (Hartmann Analytic GmbH, Braunschweig, Germany) using the chloramine-T method [28]. Free ^125^I was separated by preparative HPLC as previously described by Schieck et al. [22]. Approximately 2 to 4 MBq of the radiolabeled peptides was injected intravenously into the tail vein and images were recorded immediately post injection and after 1 h, 2 h, 3 h, 5 h, and 24 h. Scintigraphical images were obtained by using a γ-camera (Gamma Imager, Biospace Lab, Paris, France).

## 5. Conclusions

The length of the hydrophobic moiety seems to be crucial for an effective antimicrobial activity as demonstrated by the use of fatty acids with different chain lengths. Here, fatty acids of medium length (C10:0–C12:0) were found to be the most promising tool for the design of antibacterial peptides. By this modification technique, the peptide C11-Pep19-short showed improved characteristics while retaining or even increasing its antimicrobial activity. 

## Supplementary Materials

The following are available online at https://www.mdpi.com/2079-6382/9/12/844/s1, Figure S1: The HPLC/MS analysis of the peptide Pep19-short is shown. Figure S2: The HPLC/MS analysis of the peptide C11-Pep19-short is shown. Figure S3: The HPLC/MS analysis of the peptide Pep19-4LF is shown. Figure S4: The HPLC/MS analysis of the peptide C_11_-Pep19-4LF is shown. Figure S5: The HPLC/MS analysis of the peptide Pep19-2.5 is shown. Figure S6: HPLC/MS analysis after incubation of C_11_-Pep19-short with S9 mix from human liver. Figure S7: Radio-HPLC diagrams of ^125^I-labeled Pep19-4LF and C11-Pep19-short. Figure S8: MIC values of bacitracin and Pep19-short conjugated to different fatty acids. Figure S9: MIC-values for N-terminal- and C-terminal-modified C11-Pep19-short. Figure S10: Time–kill curves on *Rothia kristinae* (DSM 20032) for C11-Pep19-short, Pep19-4LF, and bacitracin at a concentration of 2 × MIC and 1 × MIC. Table S1: Calculated amount of peptide fragments after incubation of C11-Pep19-short with S9 mix from human liver. Table S2: Results of MIC and MBC studies on the Gram-negative *Acinetobacter bohemicus* (DSM 100419). Table S3: Results of MIC and MBC studies on the Gram-positive *Rothia kristinae* (DSM 20032).
