# Supplementary material for: Fatty Acid Conjugation Leads to Length-Dependent Antimicrobial Activity of a Synthetic Antibacterial Peptide (Pep19-4LF)
Source: Antibiotics (Basel). 2020 Nov 26;9(12):844. doi: 10.3390/antibiotics9120844 (PMC7760514; doi:10.3390/antibiotics9120844)
Supplement: Supplementary file 1 [file antibiotics-09-00844-s001.pdf]

antibiotics

Article

Fatty Acid Conjugation Leads to Length-Dependent Antimicrobial Activity of a Synthetic Antibacterial Peptide (Pep19-4LF)

Philip Storck [1], Florian Umstätter [1], Sabrina Wohlfart [1], Cornelius Domhan [2], Christian Kleist [1], Julia Werner [1], Klaus Brandenburg [3], Stefan Zimmermann [4], Uwe Haberkorn [1], Walter Mier [1] and Philipp Uhl [1,*]

[1] Department of Nuclear Medicine, Heidelberg University Hospital, 69120 Heidelberg, Germany; philip.storck@med.uni-heidelberg.de (P.S.); florian.umstaetter@med.uni-heidelberg.de (F.U.); sabrina.wohlfart@med.uni-heidelberg.de (S.W.); christian.kleist@med.uni-heidelberg.de (C.K.); julia.werner@med.uni-heidelberg.de (J.W.); uwe.haberkorn@med.uni-heidelberg.de (U.H.); walter.mier@med.uni-heidelberg.de (W.M.)

[2] Institute of Pharmacy and Molecular Biotechnology, Ruprecht-Karls-University, 69120 Heidelberg, Germany; Domhan@uni-heidelberg.de (C.D.)

[3] Brandenburg Antiinfektiva GmbH, Parkallee 10b, 23845 Borstel, Germany; kbrandenburg@fz-borstel.de (K.B.)

[4] Department of Infectious Diseases, Medical Microbiology and Hygiene, Heidelberg University Hospital, 69120 Heidelberg, Germany; stefan.zimmermann@med.uni-heidelberg.de (S.Z.)

* Correspondence: philipp.uhl@med.uni-heidelberg.de; Tel.: +496221567726

Supplementary Data

Refers to: 4.1 Synthesis of peptide conjugates

HPLC/MS analysis of all purified peptides was performed (for Pep19-short see figure S1).

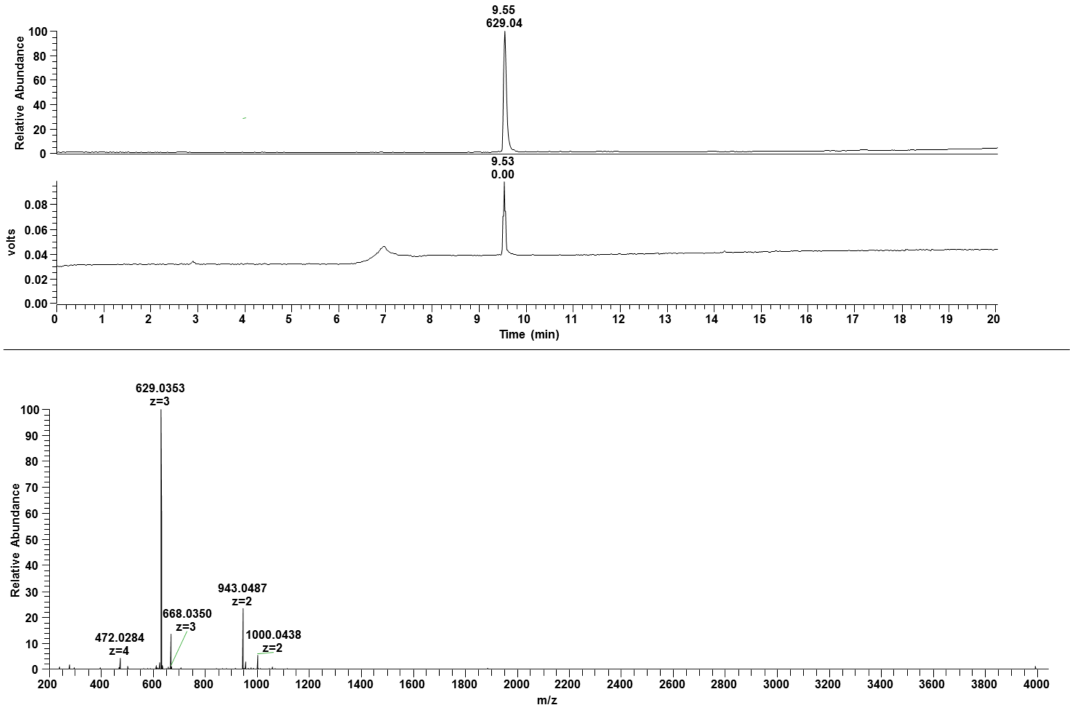

Figure S1. The HPLC/MS analysis of the peptide Pep19-short is shown.

The main signal of the mass spectrum was observed at m/z = 629.04 (z = 3). This corresponds to the peak of Pep19-short (molecular weight = 1884.13).

HPLC/MS analysis of the purified C_{11}-Pep19-short is shown in figure S2.

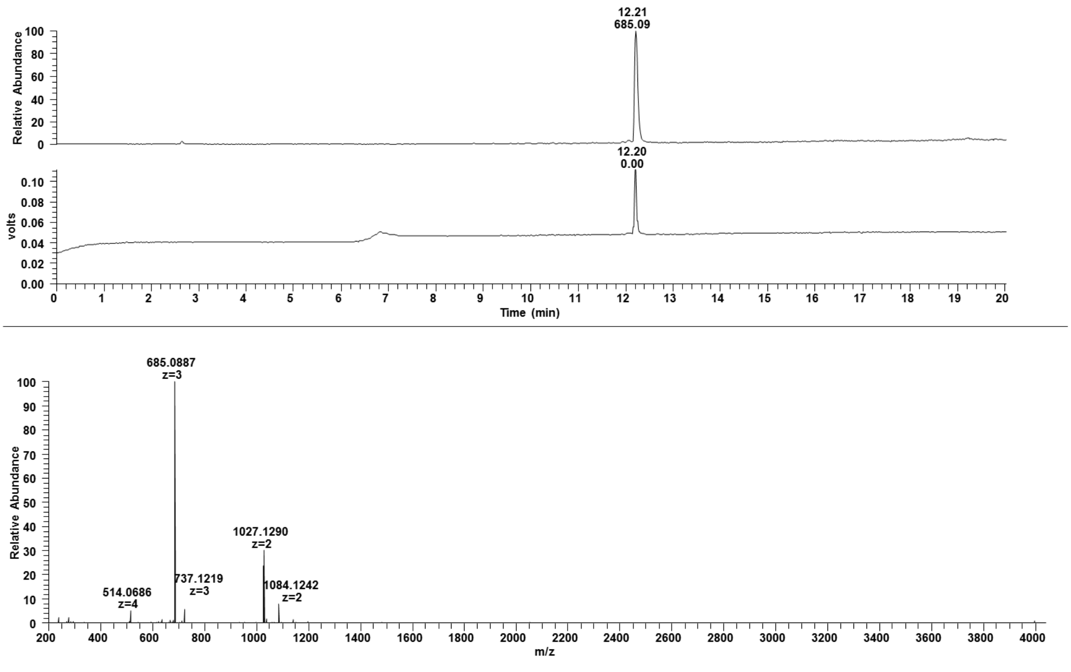

Figure S2. The HPLC/MS analysis of the peptide C_{11}-Pep19-short is shown.

The main signal of the mass spectrum was observed at m/z = 685.09 (z = 3). This corresponds to the peak of C_{11}-Pep19-short (molecular weight = 2052.43).

HPLC/MS analysis of the purified Pep19-4LF is shown in figure S3.

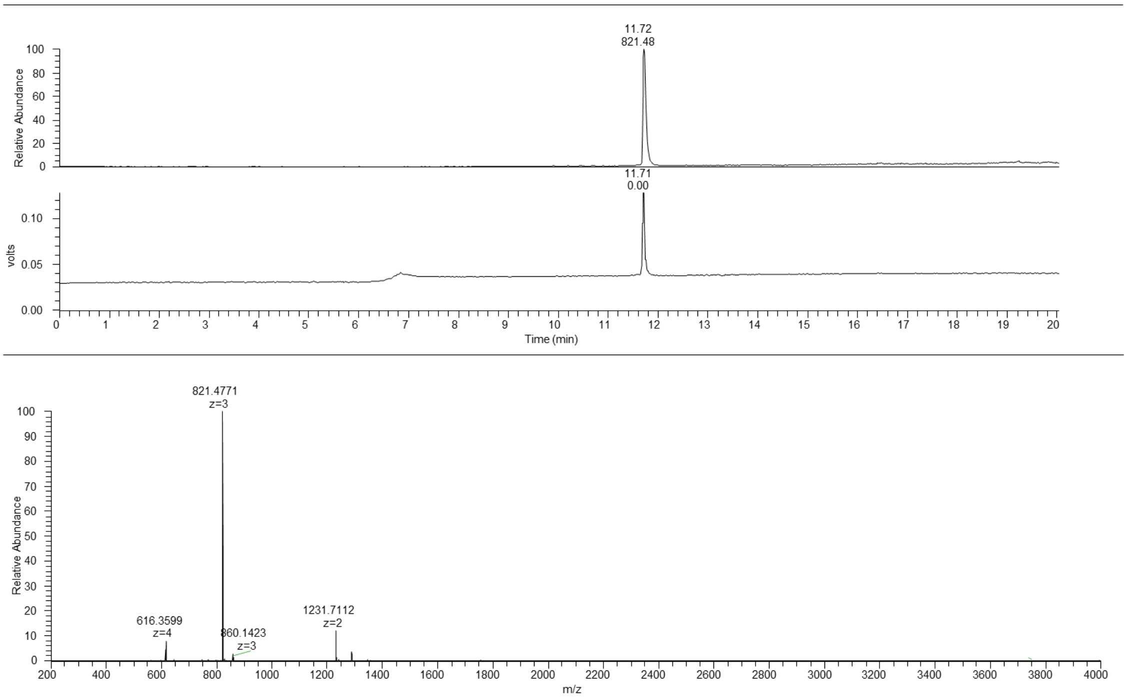

Figure S3. The HPLC/MS analysis of the peptide Pep19-4LF is shown.

The main signal of the mass spectrum was observed at m/z = 821.48 (z = 3). This corresponds to the peak of Pep19-4LF (molecular weight = 2463.02)

HPLC/MS analysis of the purified C_{11}-Pep19-4LF is shown in figure S4.

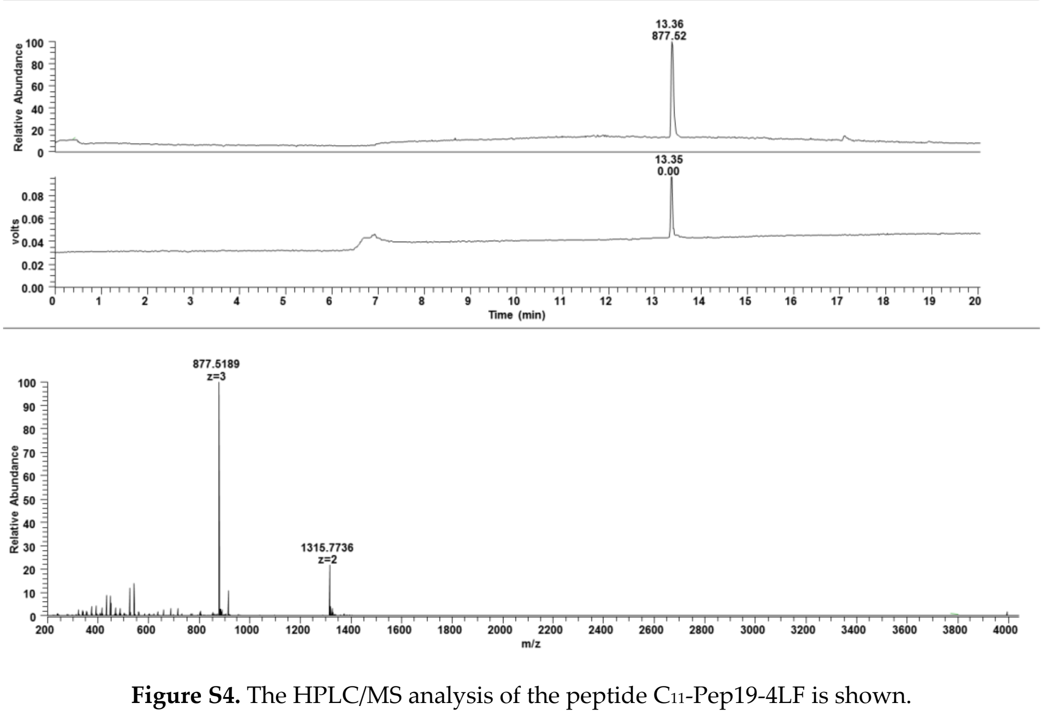

Figure S4. The HPLC/MS analysis of the peptide C_{11}-Pep19-4LF is shown.

The main signal of the mass spectrum was observed at m/z = 877.52 (z = 3). This corresponds to the peak of C_{11}-Pep19-4LF (molecular weight = 2629.76).

HPLC/MS analysis of the purified Pep19-2.5 is shown in figure S5.

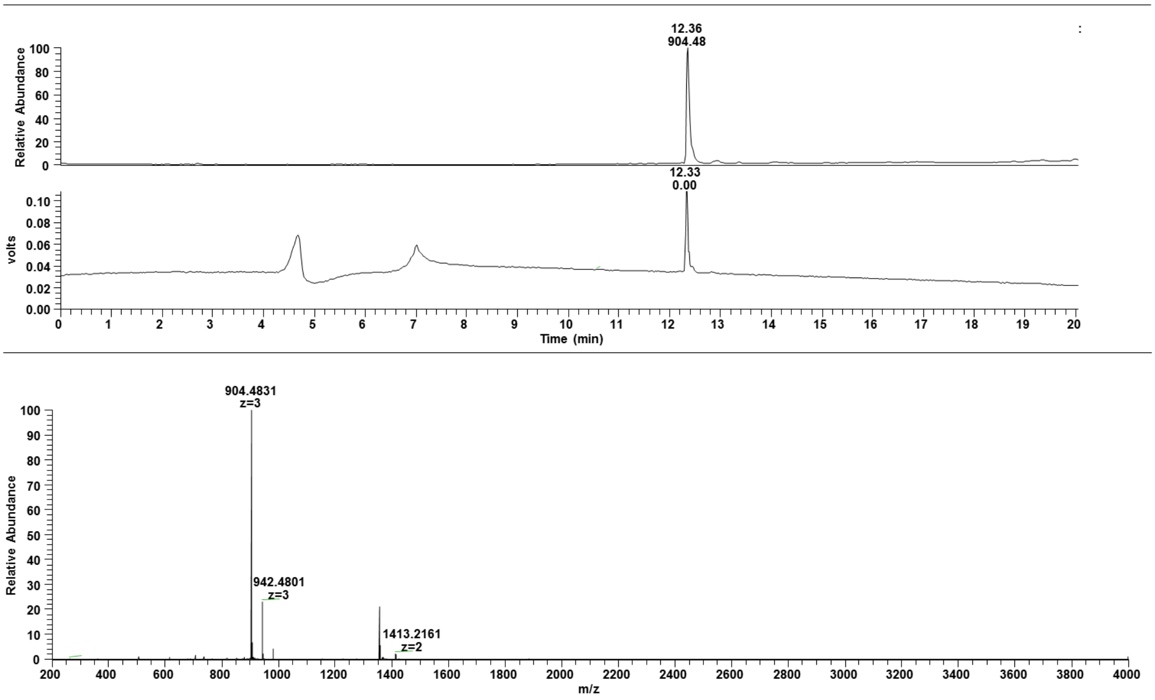

Figure S5. The HPLC/MS analysis of the peptide Pep19-2.5 is shown.

The main signal of the mass spectrum was observed at m/z = 904.48 (z = 3). This corresponds to the peak of Pep19-2.5 (molecular weight = 2710.46).

Refers to 4.4: Digestion of C11-Pep19-short with S9 fraction from human liver

Table S1. Calculated amount of peptide fragments after incubation of C11-Pep19-short with S9 mix from human liver.

Detected Mass [g/mol]	Corresponding Amino Acid Sequence	Calculated Amount [%]
685.1 [z = 3]	C11-GKKYRRFRWKFKGK (intact Peptide)	59.3
642.3 [z = 3]	C11-GKKYRRFRWKFKG	13.6
796.9 [z = 2]	C11-GKKYRRFRWK	15.6
488.7 [z = 2]	C11-GKKYRR	4.7
410.2 [z = 2]	C11-GKKYR	6.8

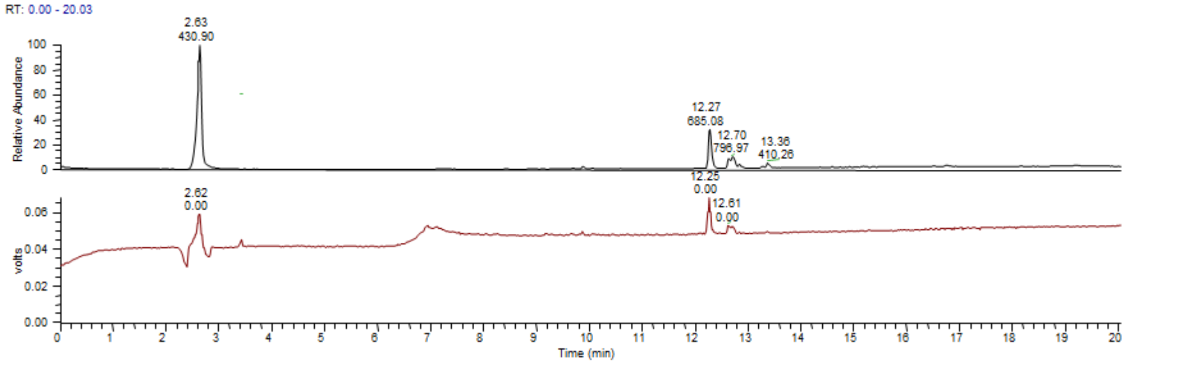

Figure S6. HPLC/MS analysis after incubation of C11-Pep19-short with S9 mix from human liver. After one hour of incubation, about 60 % of the entire peptide was still intact.

Refers to: 4.5: In vivo experiments in female Wistar rats

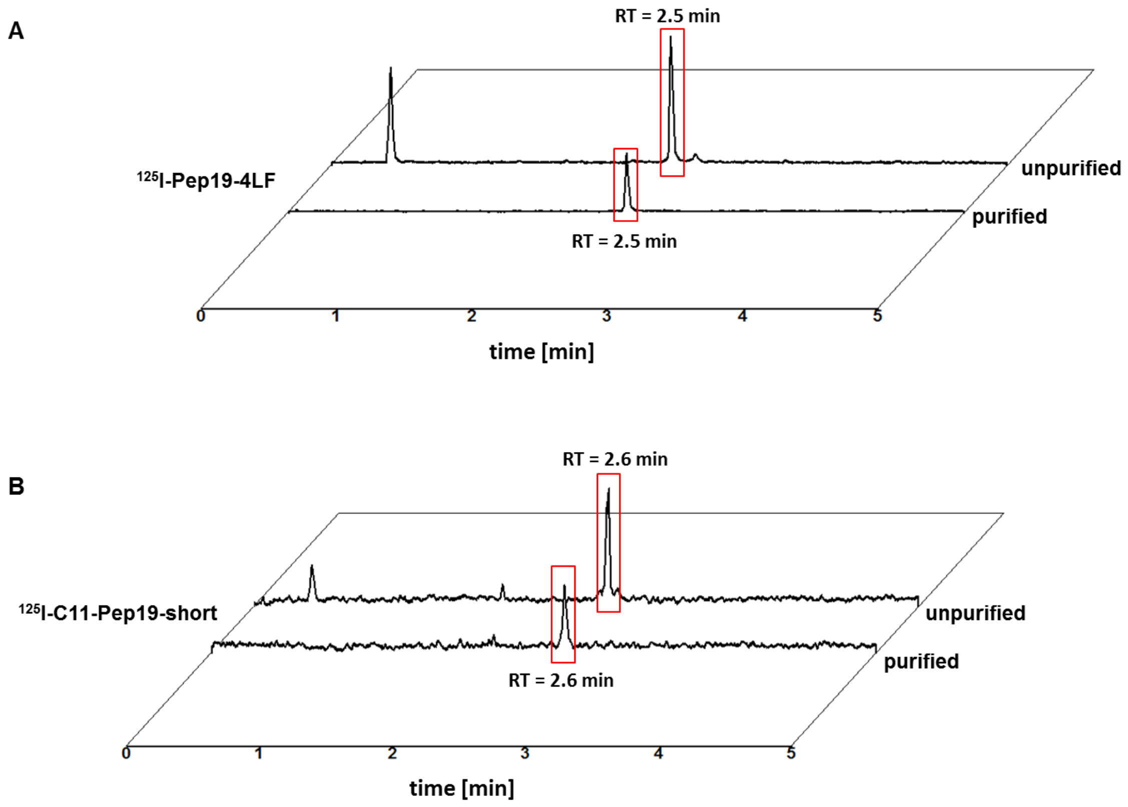

Figure S7. Radio-HPLC diagrams of ^{125}I-labeled (A) Pep19-4LF and (B) C11-Pep19-short.

Refers to: 3.1: Antibacterial activity

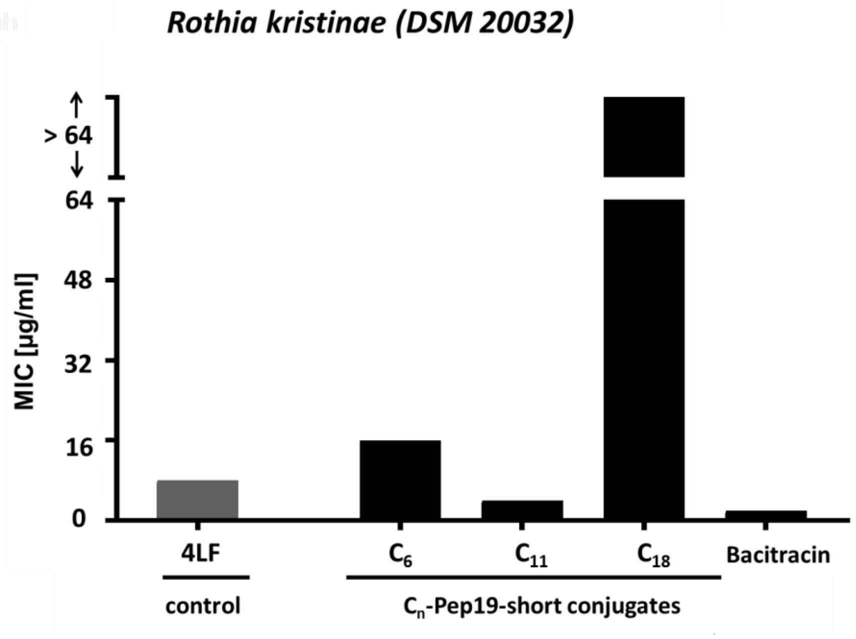

Figure S8. MIC values of Bacitracin and Pep19-short conjugated to different fatty acids. The MIC values were determined for the subsequent MBC and time-kill studies (n=3).

Table S2. Results of MIC and MBC studies on the gram-negative *Acinetobacter bohemicus* (DSM 100419) (n=3).

Conjugate	MIC value	MBC value
C_6-Pep19-short	16	16
C_8-Pep19-short	8	8
C_{10}-Pep19-short	4	4
C_{11}-Pep19-short	4	4
C_{12}-Pep19-short	4	4
C_{14}-Pep19-short	8	8
C_{16}-Pep19-short	> 64	> 64
C18-Pep19-short	> 64	> 64
Pep19-short	64	64
Pep19-4LF	8	8
Pep19-2.5	> 64	> 64

All MIC values were comparable to the MBC values indicating a bactericidal mode of action of the peptide conjugates.

Table 3. Results of MIC and MBC studies on the gram-positive *Rothia kristinae* (DSM 20032) (n=3).

Conjugate	MIC value	MBC value
C_6-Pep19-short	16	32
C_{11}-Pep19-short	4	4
C_{18}-Pep19-short	> 64	> 64
Pep19-4LF	8	8

The obtained MIC values were comparable to the MBC values indicating a bactericidal mode of action of the peptide conjugates. Only C_6-Pep19-short showed a discrepancy between MIC and MBC in the range of one dilution step. This can be explained by a fluctuation of the test itself.

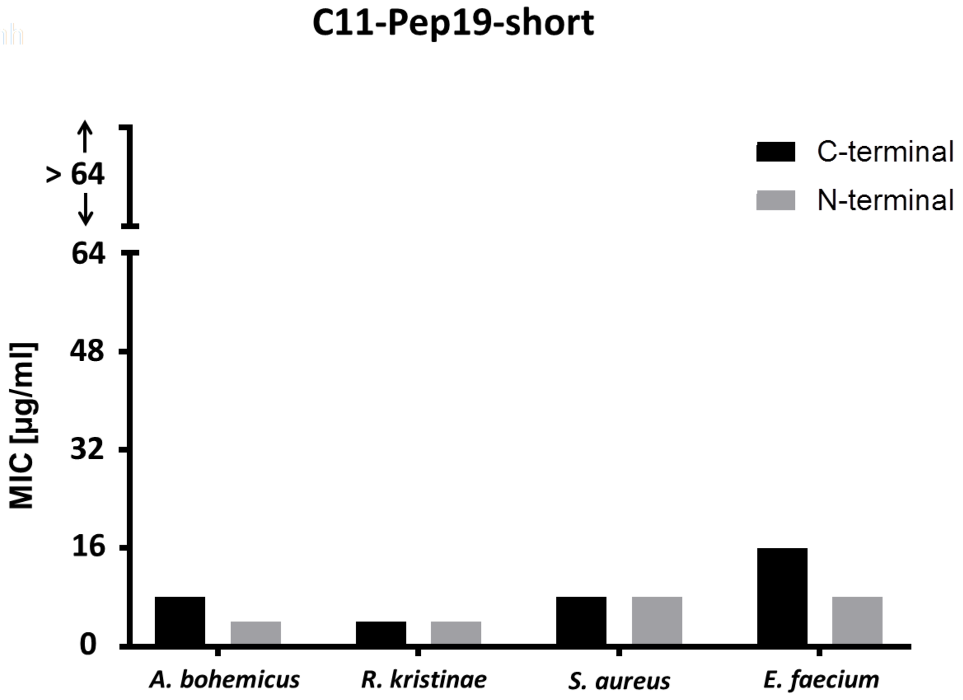

Figure S9. Comparison of MIC-values for N-terminal and C-terminal modified C11-Pep19-short. Here, undecanoic acid (C11:0) was either located at the N-terminal or C-terminal side of the peptide. The results were comparable compared to the N-terminal variant of C11-Pep19-short on all tested strains. Thus, undecanoic acid can coupled to the N-terminal side, achieving low MICs and a simplified peptide synthesis.

Time-kill study

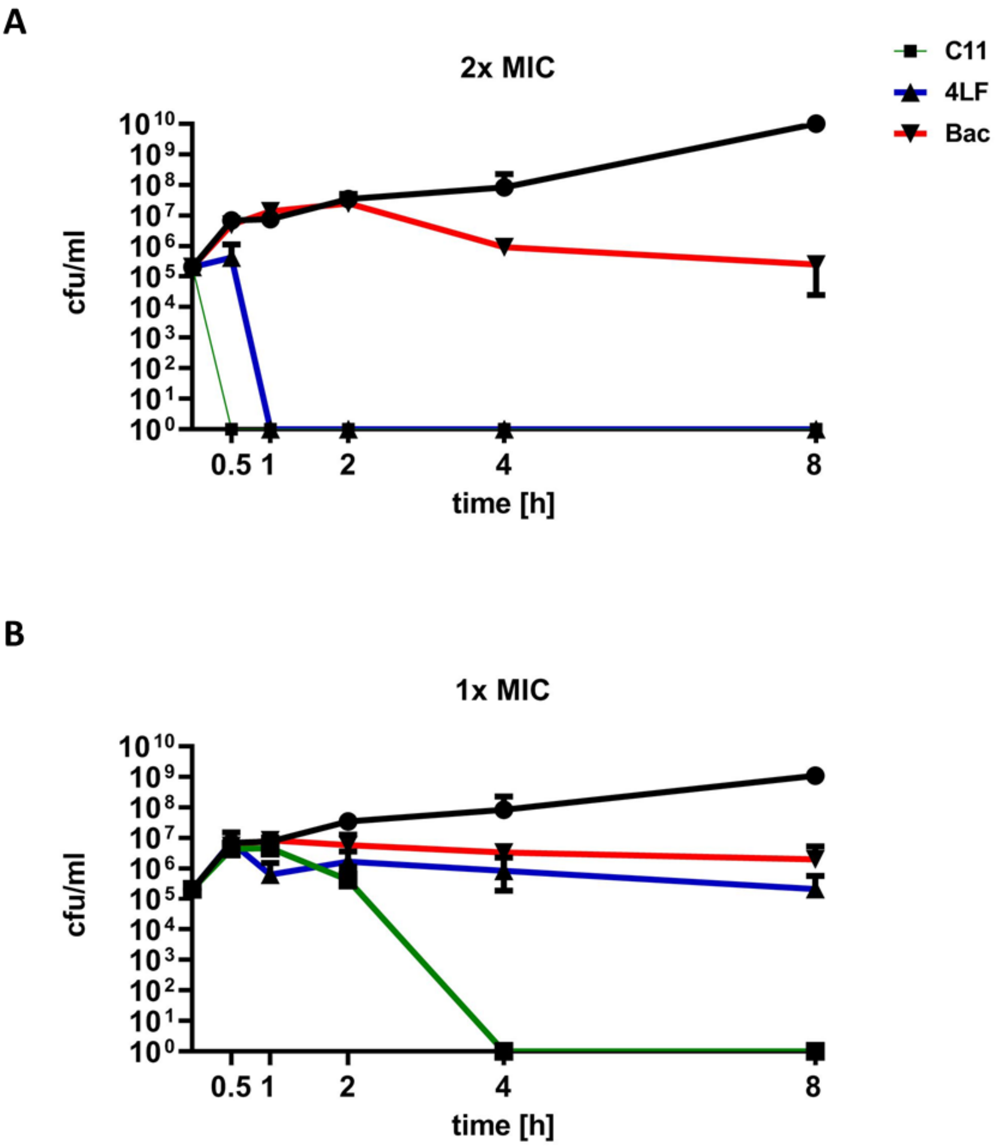

Figure S10. Time-kill curves on *Rothia kristinae (*DSM 20032) for C_{11}-Pep19-short, Pep19-4LF and Bacitracin at a concentration of 2 × MIC (A) and 1 × MIC (B) (n=3). For a concentration of 2 × MIC all bacteria were killed by C_{11}-Pep19-short after 30 minutes and by Pep19-4LF after 1 h. Bacitracin showed a bacteriostatic mode of action at this concentration with slowly decreasing numbers of cfu/ml. For a concentration of 1 × MIC, all bacteria were killed by C_{11}-Pep19-short after 4 hours. However, in this case, the concentration was not high enough to kill all bacteria with Pep19-4LF within 8 hours.
